# Allelopathic Responses of Rice Seedlings under Some Different Stresses

**DOI:** 10.3390/plants7020040

**Published:** 2018-05-08

**Authors:** Tran Dang Khanh, La Hoang Anh, La Tuan Nghia, Khuat Huu Trung, Pham Bich Hien, Do Minh Trung, Tran Dang Xuan

**Affiliations:** 1 Agricultural Genetics Institute, Pham Van Dong, Tu Liem, Hanoi 123000, Vietnam; tdkhanh@vaas.vn (T.D.K.); hoanganh6920@gmail.com (L.H.A.); khuathuutrung@yahoo.com (K.H.T.); 2 Graduate School for International Development and Cooperation (IDEC), Hiroshima University, Higashi-Hiroshima 739-8529, Japan; 3 Plant Resource Center, An Khanh, Hoai Duc, Hanoi 152900, Vietnam; latuannghia@agi.vaas.vn; 4 Post Graduate Training Department, Vietnam Academy of Agricultural Sciences, Hanoi 128200, Vietnam; bichhienvaas@gmail.com; 5 Institute of Biomedical and Pharmaceutical Applied Research Centre, Vietnam Military Medical University (VMMU), Hanoi 150000, Vietnam; dominhtrungut@yahoo.com

**Keywords:** allelopathy, allelochemical, rice, environmental stress, barnyardgrass, weeds, phenolic acids

## Abstract

The objective of this study was to evaluate the allelopathic responses of rice seedlings under submergence stress at different temperatures (10, 25, 32, and 37 °C). The results showed that a wide range of allelopathic responses of rice seedlings depended on varieties and stress conditions, with temperature was being a key factor. It showed that the extracts of rice seedlings induced significant suppression on lettuce and radish seedling germination, but had negligible allelopathic effects on growth of barnyardgrass, whilst the emergence and growth of natural weeds was stimulated. In contrast, the root exudates of Koshihikari rice seedlings (K32) at 32 °C reduced the number of total weeds by ≈60.0% and the total dry weight of weeds by 93.0%; i.e., to a greater extent than other root exudates. Among the 13 identified phenolic acids, *p-*hydroxybenzoic, vanillic, syringic, sinapic and benzoic acids—at concentrations of 0.360, 0.045, 3.052, 1.309 and 5.543 μg/mL might be involved in allelopathic responses of K32, inhibiting the growth of barnyardgrass and natural weeds. Findings of the present study may provide useful information on allelopathic responses of rice under environmental stresses and thus further understand of the competitive relationships between rice and weeds under natural conditions.

## 1. Introduction

Allelopathy is defined as chemical interaction(s) between plants that interfere with plant growth, mediated by the release of plant-produced bioactive secondary metabolites, referred to allelochemicals [1]. Allelochemical production represents a form of chemical warfare between plants competing for light, water, nutrients and other resources [2,3]. Numerous allelochemicals derived from plants have been described to stimulate and/or inhibit other species’ germination and growth. Intense scientific efforts have focused in the past on describing the mechanisms used by plants to self-regulate their own and other species’ densities and distribution or those found at the basis of biological invasions via allelopathic interactions [4,5]. Allelopathic crop varieties can release their own “phytotoxins” as allelochemicals to reduce the growth of weeds, thus permitting ecological weed management in cropping systems [6,7].

Rice (*Oryza sativa* L.) is the major food crop in Asia, providing daily sustenance to half of the global population [8]. However, under the current pressures of rapid global population growth and adverse climate change, increased attention should be directed towards new approaches in improving both crop quality and yield for sustainable rice production. For more than four decades, substantial research effort has been invested in studying rice allelopathy, finding that allelopathic activity of rice is variety and origin dependent. For example, Japonica rice shows stronger allelopathic properties than Indica or Japonica–Indica hybrid [9]. Numerous growth inhibitor allelochemicals belonging to the group of phenolic acids, fatty acids, diterpenoids, momilactones and others have been reported [10,11,12].

In nature, flowering plants in general, rice included, are faced with a plethora of antagonists, and have evolved myriad defense mechanisms by which they are able to deal with various kind of abiotic and biotic stresses [13]. Therefore, the level of allelopathic activity and allelochemical concentration plants release into the environment, including root exudates, are species specific and dependent on biotic and abiotic stress factors [1,14]. In particular, climate change is expected to adversely affect allelochemical production, and has aroused considerable interest in this area of research in recent years [15,16,17]. Previous studies have reported that in plants, produced allelochemicals, such as phenolic acids or other compounds upon biotic or abiotic stress exposure, such as cold, heat, submergence, and others are produced for protection and survival of the species under field conditions [12,18,19].

Although the numerous studies focusing on rice allelopathy mechanisms and their agricultural relevance, the allelopathic responses of rice under the specific abiotic stresses are poorly understood. Therefore, the objectives of this study were to assess the allelopathic responses of the extracts and root exudates of rice seedlings under complete submergence and temperature stress. The results of this study provide useful information for better understanding the allelopathic responses of rice seedlings under multiple stresses, expected to occur concurrently in a changing climate.

## 2. Materials and Methods

### 2.1. Rice Seeds and Indicator Plants

Seeds of two rice varieties, Koshihikari (Japonica) and Jasmine (Indica) were kindly provided by the Laboratory of Plant Physiology and Biochemistry, Graduate School for International Development and Cooperation (IDEC), Hiroshima University, Japan. Seeds of lettuce (*Lactuca sativa* L.) and radish (*Raphanus sativus* L.) were used as indicator plants because they are sensitive to allelochemicals at low concentrations [20]. Barnyardgrass (*Echinochloa crus-galli* L.) seeds were provided by Department of Genetic Engineering, Agricultural Genetics Institute, Vietnam. The germination rate of indicator plants was randomly tested in distilled water before conducting experiment and showed >95%.

### 2.2. Paddy Soils Preparation

The paddy soils (pH: 6.3, total C: 2.22%, total N: 0.18%, CEC: 8.8 meq per 100 g soil; CaO: 91, MgO: 13, K_2_O: 17, K_2_O_5_: 18, SiO_2_: 25 mg per 100 g soil) were collected randomly from the experimental farm of Hiroshima University, Hiroshima city, Japan, where early-matured rice (Koshihikari variety) had been grown. The soils were collected to a depth of 10 cm in summer 2015. After that, the soils were dried and mixed until treatment was conducted.

### 2.3. Phenolic Standards and Reagents

The standard compounds for validating the phenolic compounds, including gallic acid, protocatechuic acid, catechol, chlorogenic acid, *p-*hydroxybenzoic acid, vanillic acid, caffeic acid, syringic acid, vanillin, ferulic acid, sinapic acid, *p-*coumaric acid, benzoic acid, ellagic acid, and cinnamic acid, were purchased from Kanto Chemical Co., Inc., Tokyo, Japan.

### 2.4. Other Chemicals

The collected extracts and root exudates of rice seedlings were diluted by 1.0% Dimethyl sulfoxide (DMSO) in distilled water. Before carrying out experiments, the treatments using DMSO 1.0% were applied to evaluate the effect of DMSO 1.0% on the germination and growth of indicator plants and natural weeds. The control treatment consisted of distilled water only. The results showed that DMSO 1.0% had no effect on indicator plants and natural weeds to compare with the controls.

### 2.5. Extracts and Root Exudates of Rice Seedlings under the Stress Conditions

The seeds of two rice varieties (Koshihikari and Jasmine) were germinated in seed beds filled with moist sterilized soil for 7 days. After that, the seedlings of each variety were placed into a test tube (five individuals of each variety per tube) and were treated in complete submergence [21] and placed in dark conditions at the different specific temperatures (10, 25, 32, 37 °C) for 7 days. After that, the rice seedlings and water in each tube were collected to obtain extracts and root exudates for further experiments. A total of eight treatments with six replications were implemented in this study. All the treatments are shown in Table 1.

The extracts were obtained by grinding fresh rice seedlings and stirring in 80% MeOH + 0.1% HCl (20 mL) for 24 h at room temperature. After that, the extracts were centrifuged at 10,000 rpm for 10 min at 10 °C, followed by filtration. The combined supernatants were dried under vacuum in a rotary evaporator (SB-350-EYALA, Tokyo, Japan) at 35 °C. The obtained dried extracts were dissolved in 1.0% dimethyl sulfoxide (DMSO) with the concentration of 10 mg/mL. Finally, they were diluted with distilled water to adjust the concentration to 1 mg/mL for allelopathic evaluation against the indicator plants and paddy weeds.

The root exudates of rice seedlings were attained from the water of all the treatments, which were then extracted by using 70% EtOH (200 mL) for 10 min at room temperature, followed by filtration. Subsequently, the solvent was removed using a rotary evaporator (SB-350-EYALA, Tokyo, Japan) at 40 °C. The root exudates were dissolved in 1.0% DMSO to 10 mL and kept at 4 °C for further experiments. After that, they were diluted by 50% using distilled water to evaluate the allelopathic activity against the indicator plants and paddy weeds.

### 2.6. Allelopathic Response of the Extracts and Root Exudates from Rice Seedlings

#### 2.6.1. Evaluating Allelopathic Response of the Extracts and Root Exudates in Laboratory Condition

Allelopathic effects on the germination and growth of indicator plants, lettuce, radish and barnyargrass in the laboratory condition were evaluated. The seeds of indicator plants were rinsed several times with distilled water. Subsequently, they were surface-sterilized by immersion in 0.1% sodium hypochlorite (NaOCl) for 30 min. Finally, they were continuously rinsed several times with distilled water. Ten seeds of each type of indicator plants were placed in a sterilized beaker, then was added by 3 mL (1 mg/mL) of solution of rice seedling extracts. Similarly, another treatment was added by 3 mL of solution (50%) of root exudates, respectively. All the treatments were placed in a growth chamber for a 16 h photoperiod at 28/25 °C day/night temperature. After 7 days, the germination rate (GR), survival rate (SR), shoot height (SH), root length (RL) and dry weight (DW) of the indicator plants were recorded. The control treatment was used with distilled water only. The inhibition or stimulation percentages of the treatments were calculated over the controls.

To assess allelopathic responses on the emergence and growth of natural weeds in paddy soils, one hundred grams of paddy soils was placed in Petri dishes (diameter: 9 cm) and saturated with distilled water. After that, 3 mL solution (1 mg/mL) of the rice seedlings-extracts were added. Similarly, 3 mL solution (50%) of root exudates-extracts were directly added to the paddy soils as the separate treatment. The controlled treatments were used with distilled water only. All experiments were conducted with at least 3 replications. All Petri dishes were placed in a growth chamber with 16 h photoperiod at 28/25 °C day/night temperature. After 20 days, the number of weeds and the weeds’ DW were determined. The inhibition or stimulation percentages of the treatments were calculated.

#### 2.6.2. Evaluating Allelopathic Responses of the Extracts and Root Exudates against the Growth of Barnyardgrass in Greenhouse

Ten healthy seeds of barnyardgrass were germinated in seed trays, which were filled with sterilized soils as described above. After 14 days of growth, 3 mL solution (1 mg/mL) of the extracts and 3 mL solution (50%) of the root exudates were directly and separately sprayed onto the barnyardgrass leaves, stems and soil surface of each treatment. The control treatments were performed with distilled water only. After 1 week, the SH (cm), RL (cm) and DW (g) of the plants were recorded. The inhibition or stimulation percentages of the treatments were determined over the controls.

### 2.7. Determination of Total Phenolic and Flavonoid Contents

The total phenolic contents of the extracts and root exudates were determined by using the Folin-Cicalteau method of Medini et al. [22]. The solutions were mixed, including 0.125 mL of the extracts or root exudates, 0.5 mL of distilled water and 0.125 mL of 10% Folin-Ciocalteu reagent (in distilled water). After 6 min, an amount of 1.25 mL of 7.5% aqueous Na_2_CO_3_ solution (in distilled water) was added. Subsequently, distilled water was added for adjustment to final volume (3 mL). The absorbance was recorded at 760 nm by using spectrophotometer after incubation for 30 min at room temperature. Finally, the milligram of gallic acid equivalent (GAE)/mL was identified as an expression of total phenolic content.

The total flavonoid contents were determined using the method described by Bueno-Costa et al. [23], with some modifications. The extracts or root exudates (0.5 mL) were mixed with 0.5 mL of 2% aluminum chloride solution (in MeOH). The solutions were incubated at room temperature for 15 min, the absorption was measured at 430 nm by using spectrophotometer. Subsequently, the milligram of rutin equivalent (RE)/mL was determined as the expression of total flavonoid content.

### 2.8. Identification of Phenolic Contained in the Extracts and Root Exudates under the Stress Conditions

Phenolic profiles in the extracts and root exudates were analyzed following to the HPLC method of Xuan et al. [24]. A HPLC system (JASCO, Tokyo, Japan) consisting of a LC-Net II/ADC, UV-2075 Plus and PU-2089 Plus detector, including RPC18 column (250 mm × 4.6 mm × 5 µm), was employed for the separation of phenolic constituents. The column temperature was 25 °C. An amount of 5 µL extracts or exudates were injected after filtration through a 0.45 µm filter membrane. The mobile phase consisted of 99.8% methanol (solvent A) and 0.1% acetic acid (*v*/*v*) (solvent B). A gradient elution was run with a 1 mL/min flow-rate using the following time gradients: 5% A (0–5 min), 20% A (5–10 min), 50% A (10–20 min), 80% A (20–30 min), 100% A (30–40 min), 40–50 (100% A) min, 5% A (50–60 min). Phenolic standards (5–100 ppm) were injected into the HPLC in an amount of 5 µL. The peaks of the samples were identified and calculated based on the retention times and peak areas of phenolic standards.

### 2.9. Statistical Analyses

The data were analyzed by one-way ANOVA and two-way ANOVA using the Minitab 16.0 software for Windows. With regard to the significant differences, the means were separated using Tukey’s test at *p* < 0.05 with three replications and are expressed as the mean ± standard errors (SE).

## 3. Results

### 3.1. Allelopathic Responses in Bioassay

#### 3.1.1. Allelopathic Responses of the Extracts on the Indicator Plants

For lettuce, the percentage of germination rate was significantly inhibited by all the samples, which ranged from 7.1% to 28.6%. Among them, the stressed treatment of K37 (37° C) showed a remarkably inhibitory effect of 28.6%. The lettuce SR decreased from 3.7% to 22.6%, compared with the control. In the case of SH, five extracts exhibited inhibition on lettuce ranging from 3.7% to 18.8%, of which the SH of lettuce was significantly decreased by the effect of the K32 sample (18.8%). Otherwise, the three remaining extracts stimulated the growth of lettuce shoots, including J10 (0.5%), J32 (4.1%) and J25 (7.7%). For RL, all the extracts showed significant suppression on the growth of lettuce roots except J25 (6.7%). The maximum inhibition rate on RL was 41.9% (K37), followed by 31.2% (J37), 24.6% (K32), 24.2% (K10), 15.8% (J32), 13.5% (J10) and 11.9% (K25) (Table 2). For DW, almost all of the extracts had slight inhibitions on lettuce, ranging from 2.4% to 16.3%, except the sample K37 (28.9%). The sample J32 had no effect, while J10 and J25 stimulated the DW of lettuce by 1.8% and 4.8%, respectively. The results of GR, SR, SH, RL and DW under the effect of extracts showed that the extracts mostly affected to the plant root elongation (21.2%) in total inhibition, followed by GR, SR, DW and SH, which were 18.8%, 10.5%, 6.9% and 3.4%, respectively. All the extracts showed AI ranging from 4.4% to 21.9%, where the strongest extract was that from K37. Table 2 indicates that the effect of the extracts on the GR and DW of lettuce depended on variety factor only.

Otherwise, the effect on SH and RL was dependent on variety, temperature and the interaction between these two factors (Table 2).

For radish, almost all samples had slight inhibitory effects on the GR and SR. The extracts of K32 revealed inhibition on GR by 38.5%, and SR was strongly reduced by the effect of K10 extracts (35.6%). Interestingly, the SH of radish was significantly stimulated, by more than 40.0%. The highest stimulation rate was 63.8%, found in the treatment with K10, and the lowest stimulation rate was 40.1%, in the treatment with K37. Otherwise, all the extracts exhibited strong inhibition on RL of radish. Remarkably, K32 extracts disclosed the greatest inhibition on RL (60.9%), followed by K37, K10, J32, K25, J37, J25 and J10, with the inhibition values 50.5%, 50.4%, 46.5%, 41.8%, 36.7%, 33.6% and 24.1%, respectively. The DW of radish was slightly decreased by the effect of all extracts, except the inhibition of K37 (27.7%) and K32 (27.4%). Generally, the germination and growth of radish were suppressed by the effect of almost all extracts; AI ranged from 3.0% to 17.9%. The maximum AI inhibition rate was found in K32 extract (17.9%), followed by K37 (15.1%). There was only one sample, J10, promoting radish growth by 3.8%. The data in Table 2 indicate that only the SH of radish was significantly increased by the extracts. Meanwhile, GR, SR, RL and DW decreased, whereby all the extracts mostly affected the growth of radish roots. At *p* < 0.05, the effect of extracts on GR, DW and SH of radish was based on temperature factors. The effect on SR was not determined by either variety factor or temperature factor; however, it depended on the interaction between these two factors. The two factors included variety and temperature and the interaction between these two factors were the cause of radish RL inhibition.

The results of evaluating allelopathic activity of the extracts on barnyardgrass in the laboratory indicated that GR and SR had a negligible reduction when compared with the control. In particular, the SR was shown to be 100% in almost all extracts except J37 (96.3%). Similar to radish, it showed significant stimulation on SH of barnyardgrass, ranging from 15.6% (K37) to 51.6% (J10) with a stimulation average of 36.9%. Otherwise, the RL of barnyardgrass was slightly inhibited by the extracts from 5.9% (J25) to 29.0% (K32), except the two samples K37 and J37, which stimulated the RL of barnyardgrass by 11.8%. For DW, there were extracts from K37 showing stimulation on barnyardgrass by 11.6%. However, all the other samples had inhibition on barnyardgrass ranging from 15.1% to 24.3%. The maximum inhibition rate on DW was found in K32 extracts with 24.3% of inhibition. It found that SH was significantly inhibited. Therefore, almost all extracts generally had stimulation effect on barnyardgrass in the laboratory, ranging from 0.1% (K10) to 6.2% (J37), except K25 and K32, which exhibited inhibition rates of 2.7% and 6.2%, respectively. Interestingly, the extracts from K37 showed significant inhibition on lettuce (21.9%) and radish (15.1%), but stimulation on barnyardgrass (5.8%) in the laboratory (Table 2). Both factors of variety and temperature determined the effect of extracts on SH of barnyardgrass in laboratory, while the interaction between these two factors played no role. The effect of extracts on RL and DW of barnyardgrass was noted by variety and temperature and the interactions between these two factors.

In general, most of the extracts had inhibitory effects on the growth factors of lettuce and radish, especially RL. Interestingly, SH of radish was significantly increased by the effects of all the extracts, but RL was strongly decreased. Similar to radish, SH of barnyardgrass was stimulated, but RL was slightly reduced. Generally, most extracts had stimulation effects on barnyardgrass in germination stage. The variety and temperature factors and the interaction of these two factors had various effects on the changes of allelopathic activity of extracts against indicator plants, which was analyzed by using two-way ANOVA.

#### 3.1.2. The Effects of Root Exudates on Indicator Plants

For lettuce, there were root exudates from three samples showing negligible inhibition on GR (3.4%), including K10, J10, K25 and J37. The two samples J25 and J32 had slight stimulation on GR, by 3.4%, while samples K32 and K37 had no effect on GR, only three samples, K10, J10 and K25, exhibited insignificant inhibition, at 3.3%, 3.7% and 3.3%, respectively.

All the remaining samples had no effect on SR of lettuce. For SH, all samples had a stimulation effect, ranging from 10.1% to 39.6%, with the highest stimulation rate found in K37 (39.6%), followed by K32 (35.6%). The greatest inhibition on RL of lettuce was found in K10 (22.5%) root exudates, followed by J37 (19.2%), K25 (18.3%), J32 (16.6%), J25 (16.2%) and J10 (14.2%). For the DW of lettuce, all the samples showed a stimulation effect of more than 11.4% (J32), up to a maximum of 48.5% (K37). GR, SR and RL of lettuce were insignificantly decreased by the effect of root exudates (0.9%, 1.3%, and 11.8% respectively) (Table 3). While, SH and DW of lettuce were remarkably increased by the effect of root exudates (more than 23.0%). Generally, all the root exudates showed stimulation on lettuce in laboratory, with stimulation rates ranging from 2.4% to 19.1%. The highest stimulation rate was found in K37 (19.1%), followed by K32 (12.9%). Interestingly, the root exudates from K32 and K37 stimulated lettuce, while the extracts from K32 and K37 showed inhibition on lettuce in laboratory bioassay. The effect of root exudates on SH and RL of lettuce was dependent on variety and temperature and the interaction between these two factors.

In the case of radish, root exudates generally had no effect on the GR and SR, except some samples showed a negligible inhibition of 3.3%. Interestingly, all the samples had significant stimulation on SH of radish ranging widely from 9.8% to the maximum of 89.0%. The samples had strong stimulation on SH including K37 (89.0%), K32 (44.2%), K10 (39.0%), J32 (33.7%), J37 (33.0%). Otherwise, RL was inconsiderably inhibited except the sample J32 (40.1%). For DW, there were six samples that showed inhibition on radish, including J10 (14.6%), K25 (7.7%), J25 (13.4%), K32 (6.3%), J32 (8.9%), J37 (16.0%). The other samples, including K10 and K37, exhibited stimulation on DW of radish by 5.4% and 1.4%, respectively. However, the effect of root exudates on DW of radish was insignificant. Noticeably, all the root exudates from Koshihikari, K10, K25, K32 and K37, showed stimulation of 5.4%, 0.6%, 2.1%, 13.5% of average stimulation, respectively, on radish in laboratory. Contrarily, all the root exudates from J10, J25, J32 and J37 showed inhibition by 2.5%, 2.7%, 3.7%, and 1.0% of average inhibition, respectively (Table 3). The stimulation effect of root exudates on radish SH was based on the variety and temperature and their interactions. Meanwhile, the variety factor did not play a role in inhibitory effect of root exudates on RL of radish, which was determined by temperature factor and the interaction between variety and temperature factors.

For barnyardgrass, there were four samples that had insignificant inhibition on GR, including K10 (3.6%), J25 (3.6%), and K37 (7.1%), with the highest suppression being found in J37 (10.7%). Only J32 root exudates stimulated GR by 3.6%, while the other samples had no effect. The majority of samples had a stimulation effect on the SR of barnyardgrass from 4.0% to 8.0%, but it was insignificant. The root exudates from Jasmine generally had higher stimulation (8.0%) than the root exudates from Koshihikari (4.0%). The root exudates K25 sample had no effect on SR of barnyardgrass in laboratory. For SH, only the sample K25 had stimulation effect equaling 8.2%, but it was not significant. All other samples, including K10, J10, J25, K32, J32, K37 and J37, had inhibitory effects on SH of barnyardgrass by 7.3%, 16.8%, 3.3%, 11.7%, 18.9%, 12.3% and 12.2%, respectively.

For RL, all the samples inhibited the growth of barnyardgrass roots from 16.0% to 50.0%. The greatest inhibition on RL was found in J37 root exudates (48.8%), followed by K37 (41.3%), J32 (37.0%), J10 (29.9%), K10 (25.5%), K32 (23.6%), J25 (17.3%), and K25 (16.0%). Three samples, K10, K25 and J25, exhibited slight stimulation on DW of barnyardgrass of 1.9%, 5.3% and 12.1%, respectively. The five samples showed inhibition on DW fluctuating from 11.4% to 28.2%. In general, all the samples revealed inhibition on the germination stage of barnyardgrass. The highest inhibition was found in the J37 and K37 samples of root exudates with the AI values were 17.6% and 16.0%, respectively. Only the SR of barnyardgrass was increased by the effect of root exudates, while GR, SH, RL and DW were decreased; in particular, the RL was inhibited ≈30.0% over the controls. The varieties, temperature factors and the interaction between these two factors played a major role in the effects of root exudates on SH and RL of barnyardgrass. For DW, the variety factor had no role in the allelopathic potential of root exudates, but the temperature factor and the interaction between variety and temperature showed the allelopathic effects of root exudates on DW of baryardgrass (Table 3).

Overall, SH and DW of lettuce were stimulated by the effect of root exudates from rice seedlings, while RL was negligibly inhibited. For radish, SH was significantly increased, RL and DW were slightly reduced. Therefore, the growth of lettuce and radish was generally stimulated in the germination stage. On the contrary, the growth of barnyardgrass was inhibited by the effect of root exudates under the stressed conditions. The variety and temperature factors and the interactions of these two factors had various effects on the changes of allelopathic activity of root exudates against indicator plants, which was analyzed using two-way ANOVA.

### 3.2. Allelopathic Response of Extracts and Root Exudates on the Growth of Barnyardgrass in Greenhouse

As shown in Table 4, all the extracts and root exudates had inhibitory effect on barnyardgrass, with AI ranging from 3.6% to 22.7%. The greatest inhibition was found in K10 extracts, at 22.7%. The extracts mostly decreased the DW, by 24.8%, followed by RL (19.5%) and SH (8.9%). Meanwhile, the root exudates mostly decreased the RL, by 14.7%, followed by SH (7.4%) and DW (6.5%). The effect of extracts on RL of barnyardgrass was based on the temperature factor and the interactions between temperature and variety. For DW, the effect of extracts was dependent on only temperature factor. In the case of root exudates, the temperature factor and the interactions between temperature and variety play a role in the effect on SH and RL of barnyardgrass. The variety, temperature and the interactions between these two factors were determined the allelopathic activity of root exudates on DW barnyardgrass in greenhouse.

#### Allelopathic Response of the Extracts and Exudates on the Emergence and Growth of Natural Weeds

For the extracts, only J10 had negligible inhibition on a number of monocot weeds. The other samples had no effect or even significantly stimulated the number of monocot weeds, with a maximum stimulation recorded of 28.1% (J32 extracts). The average stimulation of extracts on the number of monocot weeds was 12.1%, and caused the increase of the DW of this weed by up to 43.3% in an average stimulation. In particular, the DW of monocot weeds was significantly increased by the effect of J25 extracts (>100.0%), followed by K25 (97.7%), K32 (60.9%), J32 (47.2%), J10 (30.0%), K10 (22.4%), K37 (19.9%). For dicotyledon weeds, all the samples showed inhibition of the germination of weeds, ranging from 7.1% (K32) to 39.9% (J37). However, the DW was slightly decreased by only four samples including K10 (11.0%), K25 (8.7%), J32 (16.7%), J37 (33.1%). The other samples, J10, J25, K32, and K37, had a stimulation effect on the DW of monocot weeds by 15.6%, 47.5%, 11.4%, and 84.0%, respectively. As a result, even the number of dicotyledon weeds was decreased by 27.5% of average inhibition, but the DW still increased by 11.1% of average stimulation. In general, the number of total weeds was inhibited by 10.4%, and the DW was increased by 32.5%. Only J37 extracts had strong inhibition on germination and growth of natural weeds, at 29.6%. The other samples had a stimulation effect, especially J25 extracts with 85.1% of average stimulation. The allelopathic potential of extracts against natural weeds was determined by the variety, temperature and the interaction between these two factors (Table 5).

For the root exudates, the number of monocot weeds was significantly decreased within a range from 9.5% to 66.7% and with an average inhibition of 41.1% compared with the control, especially the root exudates from Koshihikari at 25 and 32 °C (66.7%). As a result, the DW of the monocot weeds was also significantly reduced, with an average inhibition of 86.8%. Notably, all the root exudates had a significant inhibitory effect on DW of monocot weeds of over 73.7%, up to a maximum of 94.0%. In the case of dicotyledon weeds, there were three samples with stimulation on germination, including K10, J10 and K25 (18.6%, 14.0% and 30.2%, respectively), but it was not significant. All the other samples showed an inhibition of up to 55.8%. Most of the root exudates showed inhibition on the DW of dicotyledon weeds, except J10, J32 and K37. The average inhibition of root exudates on DW of this kind of weeds was 4.2%; in particular, the root exudates from Koshihikari at 32 °C decreased the DW of dicotyledon weeds by 85.7% over the control. In general, the number of total weeds was inhibited by an average inhibition of 26.4% by the root exudates and the DW was inhibited by an average inhibition of 80.1% in comparison with the control. All the root exudates exhibited inhibition on natural weeds. In particular, K32 root exudates had the strongest alleopathic activity against natural weeds, with 76.4% inhibition, followed by J37 with 61.2% inhibition. The allelopathic potential of root exudates depended on the variety, temperature and the interactions between these two factors (Table 5).

Overall, the extracts exhibited significant stimulation of germination and DW of natural weeds by up to 85.1%. In contrast, the root exudates showed significant inhibition of natural weeds ranging from 38.3% to 76.4%. The variety and temperature factors and the interactions of these two factors had different effects on the changes of allelopathic activity of the extracts and exudates against natural weeds.

### 3.3. Determination of Total Phenolic and Flavonoid Contents of Extracts and Exudates from Rice Seedlings

**Figure 1:**
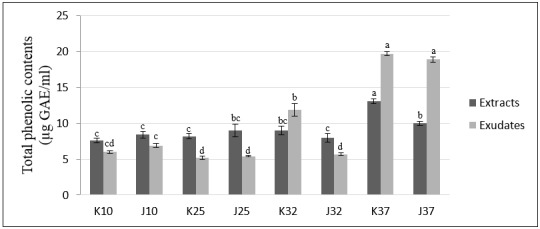
Total phenolic contents of extracts and exudates from rice seedlings affected by multiple stresses; means within the same column color followed by the different letters are significantly different at *p* < 0.05.

As shown in Figure 1, the total phenolic content of the extract from K37 was the highest (13.1 μg GAE/mL) among the extracts. The amounts of total phenolic in the other extracts, including K10, J10, K25, J25, K32, J32 and J37, were 7.6, 8.4, 8.2, 9.0, 9.0, 8.0, 10.0 μg GAE/mL, respectively. Otherwise, the total phenolic contents of the root exudates K37 and J37 were the highest (19.7 and 18.9 μg GAE/mL, respectively) among the exudates, followed by the root exudates K32 (11.9 μg GAE/mL), J10 (6.9 μg GAE/mL), K10 (6.0 μg GAE/mL), J32 (5.7 μg GAE/mL), J25 (5.4 μg GAE/mL) and K25 (5.2 μg GAE/mL). Different varieties (Koshihikari or Jasmine) resulted in different total phenolic content of the extracts. Similar to the variety factor, different temperature also had different results for the total phenolic content of the extracts. The variety and temperature factors exhibit interaction when they affect, together, the total phenolic content of the extracts. In the case of root exudates, the different varieties showed significant differences in the total phenolic content. Similarly, the temperature factor and interaction between the temperature and variety factors affected the results of the total phenolic content of root exudates.

**Figure 2:**
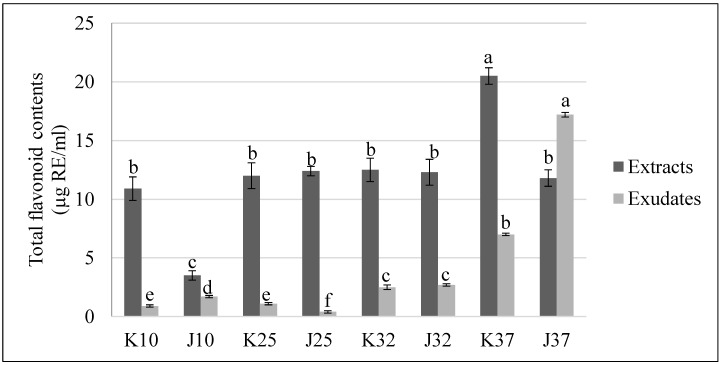
Total flavonoid contents of extracts and exudates from rice seedlings affected by multiple stresses; means within the same column color followed by the different letters are significantly different at *p* < 0.05.

As shown in Figure 2, the highest total flavonoid content was found in the K37 sample (20.5 μg RE/mL), followed by K32 (12.5 μg RE/mL), J25 (12.4 μg RE/mL), J32 (12.3 μg RE/mL), K25 (12.0 μg RE/mL), J37 (11.8 μg RE/mL), and K10 (10.9 μg RE/mL), with J10 containing the lowest (3.5 μg RE/mL). For root exudates, J37 contained the highest total flavonoid content, with 17.2 μg RE/mL, followed by K37 (7.0 μg RE/mL), J32 (2.7 μg RE/mL), K32 (2.5 μg RE/mL), J10 (1.7 μg RE/mL), K25 (1.1 μg RE/mL), and K10 (0.9 μg RE/mL), with the lowest being J25 (0.4 μg RE/mL). According to the results of data analysis using two-way ANOVA, the effects of both variety and temperature factors on the total flavonoid content of root exudates were significantly different (*p* < 0.05). In addition, the interaction between these two factors also affected the results of the total flavonoid content of root exudates from rice seedlings.

In general, rice varieties under high temperatures (37 °C) had the highest total phenolic and flavonoid contents, especially the Koshihikar rice variety.

### 3.4. Identification of Phenolic Components

Among the 15 phenolic acids, a total of 13 were detected, including gallic acid, protocatechuic acid, catechol, chlorogenic acid, *p-*hydroxybenzoic acid, vanillic acid, syringic acid, vanillin, ferulic acid, sinapic acid, *p-*coumaric acid, benzoic acid and ellagic acid. Interestingly, vanillin and benzoic acid were found in almost all extracts and root exudates. In addition, vanillic acid and syringic acid were found only in the root exudates of K32. Gallic acid was found only in the root exudates of J37 (Table 6). The strongest inhibition of natural weeds was found in the root exudates from Koshihikari at 32 °C. This sample contained five phenolic compounds in total, including *p-*hydroxybenzoic, vanillic, syringic, sinapic and benzoic acid, in the amounts of 0.360, 0.045, 3.052, 1.309 and 5.543 μg/mL, respectively.

## 4. Discussion

Weeds are major constraints, reducing global rice production. Synthetic herbicides are able to manage infestations of weeds, but also exert an adverse influence on humans and environment. Much effort has been made to control weeds by exploiting the allelopathy of rice over the last four decades [9]. More than 16,000 rice varieties collected from 99 countries have been evaluated for their allelopathic potential, and revealed that ≈4.0% of rice cultivars show weed-suppression of paddy weeds [4,25]. Some reports have shown that Japonica rice cultivars have higher allelopathic potential than Indica rice varieties [26]. Nevertheless, both Koshihikari and Jasmine cultivars have been demonstrated to be allelopathic varieties [27]. In addition, Xuan et al. [28] reported Japonica rice varieties show more abiotic tolerance than Indica rice. Notably, Khang et al. [29] demonstrated that Koshihikari was a submergence-tolerant variety, while Jasmine was a susceptible variety.

Rice is a semi-aquatic crop and is typically grown under partially flooded conditions. Hence, submergence is considered to be one of the most harmful abiotic stresses, resulting in significant losses to rice yield [30]. However, very little information is available that appraises allelopathic responses under the stress. Therefore, in this study, two rice varieties—Koshihikari (typical Japonica variety) and Jasmine (typical Indica variety)—were used to evaluate allelopathic responses, as well as to identify the correlation between allelopathic potential under the submergence and temperature stresses (10, 25, 32 and 37 °C). Rice seedlings were treated with complete submergence and different temperatures for 7 days under dark conditions. In nature, it is very difficult to distinguish allelopathic and competitive factors due to the multiple uncontrolled environment factors, which include substratum and climatic variations such as nutrients, light, moisture, temperature, etc. Therefore, in this study, rice seedlings were kept in the dark in order to accurately evaluate allelopathic responses under complete submergence and temperature stresses. In fact, dark conditions may not necessarily be a stress, as low-light shade conditions have been reported to enhance the production of allelopathic compounds [31]. Rice plants react to abiotic and biotic stresses by releasing multiple secondary metabolites to activate defense and protect themselves [32,33]. Hu et al. [34] analyzed 51 Japonica and 49 Indica rice accessions based on 121 metabolites detected by using LC-tandem, GC-MS and reported that the differences in the metabolite abundance and correlations between the two typical rice varieties may reflect the degree of metabolic adaptation and the specific phenotypes in Japonica and Indica [33]. Moreover, malic acid and long chain fatty acids of rice were enhanced under stagnant growing conditions [35].

The results of the current study reveal that the allelopathic potential of extracts and root exudates from rice seedlings depend on a wide array of interactions between the different stressed conditions. Among these, the temperature is a key factor involved in allelopathic activity. Temperature has been reported to directly influence plant growth and to possibly enhance allelochemical production, which subsequently impacts the growth of associated plants [36]. In this study, we found that the combined effect of temperature and submergence stresses was conducive to the allelopathic response, especially in allelopathic rice (Koshihikari) at 32 °C, which showed higher allelopathic activity in comparison with Jasmine. Allelopathic activity has been shown to decrease when the temperature increases to 37 °C. A plausible explanation is that Japonica samples exhibit their maximum allelopathic response under higher temperatures because of their adaptation to temperate regions with cooler climates, in contrast to the Indica sample. These results support the previous report by Xu et al. [37], who researched the allelopathic response of wild rice accessions to different temperature conditions. It can be concluded that rice seedlings are sensitive to oxygen deficiency and are markedly dependent on elevated ambient temperatures [38]. Moreover, Zobayed and Kozai [39] reported that temperature stress induced or enhanced many secondary metabolites in plants. For example, high temperature treatment improved the leaf total peroxidase activity, hypericin, pseudohypericin and hyperforin concentrations in the shoot tissues [39]. In fact, allelochemical responses may be significantly modified by environmental stress factors and may vary due to several intrinsic and extrinsic factor [40].

Phenolic compounds are important in the response of plants to biotic and abiotic stresses, for example cold, salinity, drought, etc., by determining the antioxidant activity of plants against the accumulation of reactive oxygen species (ROS) caused by stress conditions, which harm plant cells [12,41,42]. Numerous studies have reported the role of phenolic compounds in the tolerance of plants to stresses. For example, in submergence conditions, the total phenolic and flavonoid contents of rice were increased, and the occurrence of some phenolic acids probably plays a major role [29]. Another study by Weidner et al. [43] indicated that under cold stress, the levels of gallic and caffeic acids contained in extracts from germinating *Vitis californica* seeds were increased. Phenolic compounds have been also been reported to be the compounds most commonly involved in allelochemicals and responsible for allelopathic activity [9,44]. Therefore, determination of the total phenolic and flavonoid contents and the identification of the phenolic acids in extracts and root exudates from rice seedlings were performed in this study. Similar to the results of Wu et al. [45], the total phenolic and flavonoid content of the extracts of wheat (*Triticum aestivum* L.) showed an insignificant correlation with the total phenolic and flavonoid content in the root exudates.

In our study, the total phenolic and flavonoid contents were higher in the extracts and root exudates from rice seedlings under submergence and high temperature (37 °C) conditions. However, total contents of either the compounds or the released compounds from rice seedlings exhibited no correlation with allelopathic activity. These results are consistent with the study by Gautam et al. [30], reported that the amount of a particular allelochemical may be enhanced by one kind of stress and reduced by another kind of stress [46].

The identification of phenolic components showed that no particular phenolic acids were present in the samples that were strongly allelopathic against the growth of weeds. The content of individual phenolic acids in the samples that were strong against weeds was no higher than for the weak samples and was similar to the results reported by Olofsdotter et al. [11]. An explanation for this is that allelopathic potential is determined by the interaction of phenolic combinations under stresses, rather than by individual compounds. In this study, the root exudates of the sample K32 exhibited significant inhibition of natural weeds >76.0%. The allelopathic potential of this sample might be dependent on the interaction of five phenolic acids: *p-*hydroxybenzoic, vanillic, syringic, sinapic and benzoic acids, in the responded doses 0.360, 0.045, 3.052, 1.309 and 5.543 μg/mL, respectively. The study by Zeinali et al. [47] indicated that when rice cultivars are affected by salt, salicylic acid 2.0% could be an effective treatment for rice seedlings and exudates of allelochemicals against barnyardgrass. In our study, the rice variety Koshihikari under submergence and temperature stresses at 32 °C may result in exuding of *p-*hydroxybenzoic, vanillic, syringic, sinapic and benzoic acids, which possibly caused the suppression of the plants’ growth.

The obtained results indicated that this root exudation exhibited maximal allelopathic responses against barnyardgrass and natural weeds, but contrarily displayed negligible allelopathic effect on germination and growth of lettuce and radish in laboratory bioassays. This may be explained by the fact that in aquatic environments (complete submergence), nutrients, light and temperatures are important abiotic stress factors. In this study, we minimized the light and nutrients; therefore, the submergence and temperature were key factors, causing allelopathic activity as well as predisposing allelochemicals to inhibit the target indicator plants with either positive or partially antagonistic activities. The effects of allelochemicals in environments might be affected by lights, temperatures, and microbes [9,48]. The root treatments and foliar application by allelochemicals enhanced the submergence and drought tolerances in rice, respectively, have been recently demonstrated [49,50]. Therefore, further research on the correlation between allelopathic responses of plants and weeds under stress, as well as the identification of the responsible allelochemicals, should be comprehensively conducted, in order to demonstrate the mode of actions of plant defense against invasion, and utilize roles of allelochemicals in agricultural practice.

## 5. Conclusions

In summary, extracts and root exudates had the highest amount of total phenolic and flavonoid contents when rice seedlings were treated at 37 °C under abiotic stress. The allelopathic activity of extracts and root exudates of rice seedlings under the combined stresses of submergence and temperature was determined, indicating that the temperature plays a major role. Five phenolic acids—*p-*hydroxybenzoic, vanillic, syringic, sinapic and benzoic acid in the amounts of 0.360, 0.045, 3.052, 1.309 and 5.543 μg/mL, respectively—were found to be involved in the allelopathic responses of K32 root exudates, significantly inhibiting the germination and growth of barnyardgrass and natural weeds. The findings of this study may provide useful information on the correlation between abiotic stresses and the allelopathic responses of rice, leading to a better understanding of the role of the relationships between rice and weeds.

## Figures and Tables

**Table 1:** List of the samples used for treatments.

Variety	Treatment			
	10 °C	25 °C	32 °C	37 °C
Koshihikari (K)	K10	K25	K32	K37
Jasmine (J)	J10	J25	J32	J37

**Table 2:** Effects of extracts on germination of indicator plants in laboratory conditions.

Extracts	Germination Rate	Survival Rate	Shoot Height	Root Length	Dry Weight	AI
(1 mg/mL)	(%)	(%)	(cm)	(cm)	(mg)	(%)
**Lettuce**						
K10	73.3 ab (−21.4)	86.3 a (−13.7)	1.1 bc (−3.9)	2.8 cd (−24.2)	5.0 ab (−9.0)	−14.4
J10	86.7 ab (−7.1)	96.3 a (−3.7)	1.2 abc (+0.5)	3.2 b (−13.5)	5.6 a (+1.8)	−4.4
K25	73.3 ab (−21.4)	90.3 a (−9.7)	1.1 c (−7.5)	3.3 b (−11.9)	5.3 ab (−4.8)	−11.1
J25	80.0 ab (−14.3)	78.9 a (−21.1)	1.3 a (+7.7)	3.5 ab (−6.7)	5.8 a (+4.8)	−5.9
K32	70.0 b (−25.0)	95.3 a (−4.8)	0.9 d (−18.8)	2.82 cd (−24.6)	4.6 ab (−16.3)	−17.9
J32	76.7 ab (−17.9)	77.4 a (−22.6)	1.2 ab (+4.1)	3.15 bc (−15.8)	5.5 a (0.0)	−10.4
K37	66.7 b (−28.6)	95.8 a (−4.2)	1.1 c (−5.9)	2.2 e (−41.9)	3.9 b (−28.9)	−21.9
J37	80.0 ab (−14.3)	95.8 a (−4.2)	1.1 bc (−3.7)	2.6 d (−31.2)	5.4 ab (−2.4)	−11.2
Control	93.3 a (0.0)	100.0 a (0.0)	1.2 abc (0.0)	3.7 a (0.0)	5.5 a (0.0)	
AI (%)	−18.8	−10.5	−3.4	−21.2	−6.9	
P _Variety_	*	ns	*	*	*	
P _Temperature_	ns	ns	*	*	ns	
P _Variety*Temperature_	ns	ns	*	*	ns	
**Radish**						
K10	83.3 a (−3.8)	64.4 b (−35.6)	2.4 a (+63.8)	1.5 de (−50.4)	8.3 ab (−9.5)	−7.1
J10	80.0 a (−7.7)	92.6 ab (−7.4)	2.4 a (+62.4)	2.2 b (−24.1)	8.7 ab (−4.4)	+3.8
K25	73.3 ab (−15.4)	91.7 ab (−8.3)	2.4 a (+60.0)	1.7 cd (−41.8)	8.3 ab (−9.5)	−3.0
J25	76.7 ab (−11.5)	69.6 ab (−30.4)	2.3 a (+54.8)	2.0 bc (−33.6)	7.8 ab (−14.2)	−7.0
K32	53.3 b (−38.5)	88.9 ab (−11.1)	2.2 a (+48.6)	1.2 e (−60.9)	6.6 b (−27.4)	−17.9
J32	70.0 ab (−19.2)	80.8 ab (−19.2)	2.2 a (+45.9)	1.6 cde (−46.5)	7.7 ab (−16.1)	−11.0
K37	73.3 ab (−15.4)	77.8 ab (−22.2)	2.1 a (+40.1)	1.5 de (−50.5)	6.6 b (−27.7)	−15.1
J37	66.7 ab (−23.1)	90.5 ab (−9.5)	2.2 a (+50.7)	1.9 bcd (−36.7)	7.3 ab (−19.7)	−7.7
Control	86.7 a (0.0)	100.0 a (0.0)	1.5 b (0.0)	3.0 a (0.0)	9.1 a (0.0)	
AI (%)	−16.8	−18.0	+53.3	−43.1	−16.1	
P _Variety_	ns	ns	ns	*	ns	
P _Temperature_	*	ns	*	*	*	
P _Variety*Temperature_	ns	*	ns	*	ns	
**Barnyardgrass**						
K10	96.7 a (−3.3)	100.0 a (0.0)	4.5 ab (+38.4)	4.8 de (−19.3)	10.5 b (−15.1)	+0.1
J10	96.7 a (−3.3)	100.0 a (0.0)	4.9 a (+51.6)	4.6 de (−22.8)	9.9 b (−19.5)	+1.2
K25	93.3 a (−6.7)	100.0 a (0.0)	4.0 ab (+24.4)	5.0 cd (−15.9)	10.5 b (−15.1)	−2.7
J25	86.7 a (−13.3)	100.0 a (0.0)	4.7 ab (+44.6)	5.6 bc (−5.9)	10.0 b (−18.9)	+1.3
K32	93.3 a (−6.7)	100.0 a (0.0)	4.2 abc (+29.2)	4.2 e (−29.0)	9.3 b (−24.3)	−6.2
J32	96.7 a (−3.3)	100.0 a (0.0)	4.7 ab (+46.0)	4.8 de (−19.1)	9.7 b (−21.1)	+0.5
K37	90.0 a (−10.0)	100.0 a (0.0)	3.7 bc (+15.6)	6.7 a (+11.8)	13.8 a (+11.6)	+5.8
J37	93.3 a (−6.7)	96.3 a (−3.7)	4.7 ab (+45.0)	6.7 a (+11.8)	10.4 b (−15.4)	+6.2
Control	100.0 a (0.0)	100.0 a (0.0)	3.2 c (0.0)	6.0 ab (0.0)	12.3 a (0.0)	
AI (%)	−6.7	−0.5	+36.9	−11.1	−14.7	
P _Variety_	ns	ns	*	*	*	
P _Temperature_	ns	ns	*	*	*	
P _Variety*Temperature_	ns	ns	ns	*	*	

Means within a column followed by the different letters, are significantly different at *p* < 0.05. Numbers in parentheses are the rates in comparison with control; “+” symbol shows promotion percentage over control, “−” symbol shows inhibition percentage over control. AI: Average Inhibition. *: significantly different (*p* < 0.05), ns: not significantly different.

**Table 3:** Effects of the root exudates on germination of indicator plants in laboratory.

Exudates	Germination Rate	Survival Rate	Shoot Height	Root Length	Dry Weight	AI
(50%)	(%)	(%)	(cm)	(cm)	(mg)	(%)
**Lettuce**						
K10	93.3 a (−3.4)	96.7 a (−3.3)	1.3 cd (+21.4)	3.0 c (−22.5)	6.7 a (+19.8)	+2.4
J10	93.3 a (−3.4)	96.3 a (−3.7)	1.3 cd (+21.2)	3.3 bc (−14.2)	6.7 a (+19.8)	+3.9
K25	93.3 a (−3.4)	96.7 a (−3.3)	1.3 bc (+26.0)	3.2 c (−18.3)	6.6 a (+19.2)	+4.0
J25	100.0 a (+3.4)	100.0 a (0.0)	1.2 de (+10.1)	3.3 c (−16.2)	6.6 a (+18.6)	+3.2
K32	96.7 a (0.0)	100.0 a (0.0)	1.4 ab (+35.6)	4.1 a (+5.6)	6.9 a (+23.4)	+12.9
J32	100.0 a (+3.4)	100.0 a (0.0)	1.3 bc (+26.1)	3.2 c (−16.6)	6.2 a (+11.4)	+4.9
K37	96.7 a (0.0)	100.0 a (0.0)	1.5 a (+39.6)	4.2 a (+7.2)	8.3 a (+48.5)	+19.1
J37	93.3 a (−3.4)	100.0 a (0.0)	1.3 bc (+23.9)	3.1 c (−19.2)	6.9 a (+24.6)	+5.2
Control	96.7 a (0.0)	100.0 a (0.0)	1.1 e (0.0)	3.9 ab (0.0)	5.6 a (0.0)	
AI (%)	−0.9	−1.3	+25.5	−11.8	+23.2	
P _Variety_	ns	ns	*	*	ns	
P _Temperature_	ns	ns	*	*	ns	
P _Variety*Temperature_	ns	ns	*	*	ns	
**Radish**						
K10	96.7 a (−3.3)	100.0 a (0.0)	1.9 bc (+39.0)	3.9 a (−14.1)	12.3 a (+5.4)	+5.4
J10	100.0 a (0.0)	96.7 a (−3.3)	1.5 d (+11.0)	4.3 a (−5.7)	10.0 a (−14.6)	−2.5
K25	96.7 a (−3.3)	100.0 a (0.0)	1.7 cd (+20.7)	4.2 a (−6.8)	10.8 a (−7.7)	+0.6
J25	96.7 a (−3.3)	100.0 a (0.0)	1.5 d (+9.8)	4.3 a (−6.6)	10.1 a (−13.4)	−2.7
K32	100.0 a (0.0)	90.0 a (−10.0)	2.0 b (+44.2)	3.8 a (−17.6)	10.9 a (−6.3)	+2.1
J32	100.0 a (0.0)	96.7 a (−3.3)	1.8 bc (+33.7)	2.7 b (−40.1)	10.6 a (−8.9)	−3.7
K37	96.7 a (−3.3)	100.0 a (0.0)	2.6 a (+89.0)	3.7 ab (−19.5)	11.8 a (+1.4)	+13.5
J37	96.7 a (−3.3)	100.0 a (0.0)	1.8 bc (+33.0)	3.7 ab (−18.9)	9.8 a (−16.0)	−1.0
Control	100.0 a (0.0)	100.0 a (0.0)	1.4 d (0.0)	4.6 ab (0.0)	11.7 a (0.0)	
AI (%)	−2.1	−2.1	+35.1	−16.2	−7.5	
P _Variety_	ns	ns	*	ns	ns	
P _Temperature_	ns	ns	*	*	ns	
P _Variety*Temperature_	ns	ns	*	*	ns	
**Barnyardgrass**						
K10	90.0 a (−3.6)	100.0 a (+8.0)	4.4 bcd (−7.3)	6.1 cd (−25.5)	16.0 abc (+1.9)	−5.3
J10	93.3 a (0.0)	96.3 a (+4.0)	3.9 e (−16.8)	5.7 de (−29.9)	11.3 c (−28.2)	−14.2
K25	93.3 a (0.0)	92.6 a (0.0)	5.1 a (+8.2)	6.9 b (−16.0)	16.6 ab (+5.3)	−0.5
J25	90.0 a (−3.6)	100.0 a (+8.0)	4.6 bc (−3.3)	6.8 bc (−17.3)	17.6 a (+12.1)	−0.8
K32	93.3 a (0.0)	96.3 a (+4.0)	4.2 cde (−11.7)	6.2 bcd (−23.6)	12.2 bc (−22.7)	−10.8
J32	96.7 a (+3.6)	100.0 a (+8.0)	3.8 e (−18.9)	5.2 ef (−37.0)	13.9 abc (−11.4)	−11.1
K37	86.7 a (−7.1)	96.3 a (+4.0)	4.1 de (−12.3)	4.8 fg (−41.3)	12.1 bc (−23.3)	−16.0
J37	83.3 a (−10.7)	100.0 a (+8.0)	4.1 de (−12.2)	4.2 g (−48.8)	11.9 bc (−24.2)	−17.6
Control	93.3 a (0.0)	92.6 a (0.0)	4.7 ab (0.0)	8.2 a (0.0)	15.7 abc (0.0)	
AI (%)	−2.7	+5.5	−9.3	−29.9	−11.3	
P _Variety_	ns	ns	*	*	ns	
P _Temperature_	ns	ns	*	*	*	
P _Variety*Temperature_	ns	ns	*	*	*	

Means within a column followed by the different letters are significantly different at *p* < 0.05. Numbers in parentheses are the rates in comparison with control; “+” symbol shows promotion percentage over control, “−” symbol shows inhibition percentage over control. AI: Average Inhibition. *: significantly different (*p* < 0.05), ns: not significantly different.

**Table 4:** Effects of extracts and root exudates on growth of barnyardgrass in greenhouse.

Sample	Shoot Height	Root Length	Dry Weight	AI
	(cm)	(cm)	(g)	(%)
**Extracts (1 mg/mL)**				
K10	45.9 a (−5.4)	18.2 b (−39.7)	1.7 bc (−23.0)	−22.7
J10	43.5 a (−10.3)	24.3 ab (−19.4)	1.7 bc (−20.2)	−16.6
K25	42.8 a (−11.7)	26.4 a (−12.6)	1.4 c (−33.8)	−19.4
J25	43.8 a (−9.8)	25.2 a (−16.6)	1.6 bc (−28.6)	−18.3
K32	44.7 a (−7.8)	26.4 a (−12.6)	1.8 ab (−15.7)	−12.0
J32	45.5 a (−6.2)	24.3 ab (−19.4)	1.7 bc (−23.2)	−16.3
K37	42.3 a (−12.9)	24.9 ab (−17.6)	1.5 bc (−30.0)	−20.2
J37	44.9 a (−7.4)	24.6 ab (−18.4)	1.7 bc (−23.7)	−16.5
Control	48.5 a (0.0)	30.2 a (0.0)	2.2 a (0.0)	
AI (%)	−8.9	−19.5	−24.8	
P _Variety_	ns	ns	ns	
P _Temperature_	ns	*	*	
P _Variety*Temperature_	ns	*	ns	
**Exudates (50%)**				
K10	42.6 bc (−11.3)	20.0 abc (−8.4)	3.3 a (+3.8)	−5.3
J10	42.4 c (−11.8)	19.0 bcd (−13.4)	3.0 e (−5.2)	−10.1
K25	44.6 abc (−7.2)	17.3 cd (−20.9)	2.5 g (−23.0)	−17.0
J25	48.0 a (−0.2)	16.2 d (−25.9)	3.0 e (−5.1)	−10.4
K32	43.5 bc (−9.5)	18.8 cd (−14.1)	3.2 c (−0.7)	−8.1
J32	46.9 ab (−2.4)	17.3 cd (−20.9)	2.7 f (−17.1)	−13.5
K37	46.1 abc (−4.0)	18.4 cd (−16.2)	3.1 d (−3.8)	−8.0
J37	42.0 c (−12.6)	22.4 a (+2.5)	3.2 c (−0.8)	−3.6
Control	48.1 a (0.0)	21.9 a (0.0)	3.2 b (0.0)	
AI (%)	−7.4	−14.7	−6.5	
P _Variety_	ns	ns	*	
P _Temperature_	*	*	*	
P _Variety*Temperature_	*	*	*	

Means within a column followed by the different letters are significantly different at *p* < 0.05. Numbers in parentheses are the rates in comparison with control; “+” symbol shows promotion percentage over control, “−” symbol shows inhibition percentage over control. AI: Average Inhibition. *: significantly different (*p* < 0.05), ns: not significantly different.

**Table 5:** Allelopathic response of the extracts and root exudates on the emergence and growth of natural weeds.

Sample	Number of Weeds			Dry Weight of Weeds (mg)			AI
	Monocot	Dicotyledon	Total Weeds	Monocot	Dicotyledon	Total Weeds	(%)
**Extracts (1 mg/mL)**							
K10	24.3 abc (+14.1)	22.7 bc (−19.0)	47.0 a (−4.7)	21.1 cd (+22.4)	7.8 def (−11.0)	28.9 cd (+11.2)	+3.3
J10	20.7 c (−3.1)	19.0 cd (−32.1)	39.7 bc (−19.6)	22.4 cd (+30.0)	10.1 c (+15.6)	32.5 bcd (+25.1)	+2.8
K25	24.7 abc (+15.6)	19.3 cd (−31.0)	44.0 abc (−10.8)	34.1 ab (+97.7)	8.0 cdef (−8.7)	42.1 ab (+61.8)	+25.5
J25	26.0 ab (+21.9)	22.3 bc (−20.2)	48.3 a (−2.0)	35.7 a (+107.2)	12.9 b (+47.5)	48.6 a (+87.1)	+85.1
K32	21.7 bc (+1.6)	26.0 ab (−7.1)	47.7 a (−3.4)	27.7 abc (+60.9)	9.8 cd (+11.4)	37.5 bcd (+44.2)	+20.4
J32	27.3 a (+28.1)	18.0 cd (−35.7)	45.3 ab (−8.1)	25.4 bcd (+47.2)	7.3 ef (−16.7)	32.7 bcd (+25.6)	+8.8
K37	25.3 abc (+18.8)	18.0 cd (−35.7)	43.3 abc (−12.2)	20.7 cd (+19.9)	16.1 a (+84.0)	36.8 bc (+41.5)	+14.7
J37	21.3 bc (0.0)	17.0 d (−39.3)	38.3 c (−22.3)	10.5 e (−38.9)	5.9 f (−33.1)	16.4 e (−36.9)	−29.6
Control	21.3 bc (0.0)	28.0 a (0.0)	49.3 a (0.0)	17.2 de (0.0)	8.8 cde (0.0)	26.0 de (0.0)	
AI (%)	+12.1	−27.5	−10.4	+43.3	+11.1	+32.5	
P _Variety_	ns	*	*	ns	*	*	
P _Temperature_	ns	*	*	*	*	*	
P _Variety*Temperature_	*	*	*	*	*	*	
**Root Exudates (50%)**							
K10	4.3 bcd (−38.1)	17.0 ab (+18.6)	21.3 a (0.0)	2.0 de (−88.3)	1.1 cd (−21.4)	3.1 d (−83.8)	−41.9
J10	4.3 bcd (−38.1)	16.3 ab (+14.0)	20.7 a (−3.1)	3.2 bcd (−86.1)	1.8 ab (+28.6)	5.0 c (−73.4)	−38.3
K25	2.3 d (−66.7)	18.7 a (+30.2)	21.0 a (−1.6)	1.3 e (−92.7)	1.3 c (−9.5)	2.5 de (−86.5)	−42.5
J25	5.0 abc (−28.6)	10.7 c (−25.6)	15.7 b (−26.6)	1.2 e (−92.9)	1.3 c (−4.8)	2.6 de (−86.3)	−56.5
K32	2.3 d (−66.7)	6.3 e (−55.8)	8.7 d (−59.4)	1.0 e (−94.0)	0.2 e (−85.7)	1.2 e (−93.4)	−76.4
J32	4.7 bc (−33.3)	6.7 de (−53.5)	11.3 cd (−46.9)	3.5 bc (−79.7)	2.1 a (+50.0)	5.6 bc (−70.0)	−58.5
K37	6.3 ab (−9.5)	7.7 de (−46.5)	14.0 bc (−34.4)	4.6 b (−73.7)	2.1 a (+52.4)	6.7 b (−64.3)	−49.4
J37	3.7 cd (−47.6)	9.3 cd (−34.9)	13.0 bc (−39.1)	2.3 cde (−86.6)	0.8 d (−42.9)	3.1 d (−83.3)	−61.2
Control	7.0 a (0.0)	14.3 b (0.0)	21.3 a (0.0)	17.4 a (0.0)	1.4 bc (0.0)	18.8 a (0.0)	
AI (%)	−41.1	−19.2	−26.4	−86.8	−4.2	−80.1	
P _Variety_	ns	*	*	*	*	*	
P _Temperature_	*	*	*	*	*	*	
P _Variety*Temperature_	*	*	*	*	*	*	

Means within a column followed by the different letters are significantly different at *p* < 0.05. Numbers in parentheses are the rates in comparison with control; “+” symbol shows promotion percentage over control, “−” symbol shows inhibition percentage over control. AI: Average Inhibition. *: significantly different (*p* < 0.05), ns: not significantly different.

**Table 6:** Phenolic acids (μg/mL) in extracts and exudates from rice seedlings under stressed conditions.

Sample	GA	PA	Ca	ChA	*p-*HA	VA	SyA	Vn	FA	SA	*p-*CA	BA	EA
K10 (Ext)	-	-	-	-	-	-	-	2.651 cde	-	-	-	3.222 b	-
J10 (Ext)	-	-	-	-	-	-	-	2.543 def	-	1.269 b	-	2.273 bc	-
K25 (Ext)	-	0.684 a	-	-	-	-	-	2.441 f	-	1.160 b	-	-	-
J25 (Ext)	-	-	-	-	-	-	-	-	-	1.443 b	1.340 a	-	2.101 b
K32 (Ext)	-	-	-	-	0.572 ab	-	-	-	-	-	-	2.206 bc	1.665 b
J32 (Ext)	-	0.658 c	-	-	0.372 c	-	-	2.733 bcd	-	2.152 a	-	3.388 b	-
K37 (Ext)	-	-	-	6.969 a	-	-	-	-	1.285 a	-	-	1.046 c	1.825 b
J37 (Ext)	-	-	-	-	-	-	-	3.371 a	-	-	-	5.347 a	-
K10 (Exu)	-	-	-	-	-	-	-	2.907 b	-	-	-	1.109 c	-
J10 (Exu)	-	-	-	-	-	-	-	2.459 ef	-	-	1.141 b	3.102 b	-
K25 (Exu)	-	0.674 b	-	-	-	-	-	2.778 bc	-	-	-	-	-
J25 (Exu)	-	-	-	-	-	-	-	2.832 bc	-	-	-	-	-
K32 (Exu)	-	-	-	-	0.360 c	0.045 a	3.052 a	-	-	1.309 b	-	5.543 a	-
J32 (Exu)	-	-	-	-	0.620 a	-	-	2.536 def	-	-	-	1.553 c	-
K37 (Exu)	-	-	0.638 a	5.912 c	0.513 ab	-	-	2.544 def	1.198 a	-	-	-	1.871 b
J37 (Exu)	0.804 a	-	0.379 b	6.300 b	0.455 bc	-	-	2.351 f	0.976 b	2.374 a	-	-	2.871 a

Means within a column followed by the different letters are significantly different at *p* < 0.05. Ext: extracts; Exu: root exudates. GA: gallic acid, PA: protocatechuic acid, Ca: catechol, ChA: chlorogenic acid, *p*-HA: *p*-hydroxybenzoic acid, VA: vanillic acid, SyA: syringic acid, Vn: vanillin, FA: ferulic acid, SA: sinapic acid, *p*-CA: *p*-coumaric acid, BA: benzoic acid, EA: ellagic acid, -: not detected.
